# Brain MRI Analysis for Alzheimer’s Disease Diagnosis Using CNN-Based Feature Extraction and Machine Learning

**DOI:** 10.3390/s22082911

**Published:** 2022-04-11

**Authors:** Duaa AlSaeed, Samar Fouad Omar

**Affiliations:** College of Computer and Information Sciences, King Saud University, Riyadh 11451, Saudi Arabia; samar.fouad.omar@gmail.com

**Keywords:** Alzheimer’s disease, deep learning, convolutional neural network (CNN), MRI, brain imaging

## Abstract

Alzheimer’s disease is the most common form of dementia and the fifth-leading cause of death among people over the age of 65. In addition, based on official records, cases of death from Alzheimer’s disease have increased significantly. Hence, early diagnosis of Alzheimer’s disease can increase patients’ survival rates. Machine learning methods on magnetic resonance imaging have been used in the diagnosis of Alzheimer’s disease to accelerate the diagnosis process and assist physicians. However, in conventional machine learning techniques, using handcrafted feature extraction methods on MRI images is complicated, requiring the involvement of an expert user. Therefore, implementing deep learning as an automatic feature extraction method could minimize the need for feature extraction and automate the process. In this study, we propose a pre-trained CNN deep learning model ResNet50 as an automatic feature extraction method for diagnosing Alzheimer’s disease using MRI images. Then, the performance of a CNN with conventional Softmax, SVM, and RF evaluated using different metric measures such as accuracy. The result showed that our model outperformed other state-of-the-art models by achieving the higher accuracy, with an accuracy range of 85.7% to 99% for models with MRI ADNI dataset.

## 1. Introduction

The brain is one of the most significant and complex organs in the human body. It has several vital functions, such as idea formation, problem-solving, thinking, decision-making, imagination, and memory. Memory can save and retrieve information or experiences. Our physical memory stores the whole record of our lives and plays an essential role in defining our character and identity. Memory loss caused by dementia and the inability to recognize our environment are terrifying experiences. Alzheimer’s disease (AD) is the most common form of dementia. Becoming older increases people’s fears of developing Alzheimer’s. Alzheimer’s disease gradually kills brain cells and, as a result of that, patients end up disconnecting from everything around them and losing loving memories, childhood memories, the ability to recognize their family members, and even the ability to follow simple instructions. They also lose the ability to swallow, cough, and breathe in advanced stages. Approximately 50 million people worldwide are affected by dementia, and the cost of providing health and social care for them is equivalent to the world’s 18th largest economy [1]. In addition, the annual number of new cases of AD and other dementias is projected to triple by 2050, reaching 152 million cases, which means one new case of dementia every 3 seconds. Diagnosis of AD is complicated by its overlapping symptoms with normal ageing or vascular dementia (VD) [2,3]. Early and accurate diagnosis of AD plays an essential role in prevention, treatment, and patient care through tracking its development. The focus of several research projects is to detect Alzheimer’s disease using brain imaging, including MRI. It can measure the size and number of cells in the brain. Also, it can show the parietal atrophy for AD cases [4].

Images play an essential role in many scientific fields. In addition, medical imaging has become a powerful tool to understand brain functions. Brain imaging/neuroimaging, such as magnetic resonance imaging (MRI), has been used in the medical diagnosis of brain conditions to enable visualization of the structure and functionality of the brain. Physicians evaluate AD signs and symptoms, as well as perform several tests to diagnose AD dementia. Doctors may order additional laboratory tests, brain imaging tests, or memory testing for patients. These tests can help doctors make diagnoses by ruling out other conditions that cause similar symptoms. MRI can detect brain abnormalities associated with mild cognitive impairment (MCI) and can be used to predict which MCI patients will develop AD in the future. They will be looking in MRI images for any abnormalities, such as a decrease in the size of different areas of the brain (mainly affecting the temporal and parietal lobes).

With the evolution of technology and the growth of data generated by brain-imaging techniques, machine learning (ML) and deep learning (DL) are becoming increasingly crucial for extracting accurate and relevant information and making accurate predictions of AD from brain-imaging data.

Several machine-learning methods have been applied for the classification of AD, and the results of the models show good performance. In general, the conventional learning-based methods consist of three stages: 1—the predetermination of the regions of interest (ROIs) of the brain, 2—features selection from the ROIs, and 3—the classification models are built and evaluated. The main issue with conventional learning-based methods is the process of features engineering (i.e., manual selection and extraction), which has a great influence on the performance of the model. Compared with traditional ML methods, DL has become a revolutionizing methodology in recent decades [5]. Instead of extracting the features manually and in a separate process from the classifier, DL has automated the process without the engagement of human experts for feature extraction because it can learn directly from images through the neural networks. Recently, convolutional neural networks (CNNs) have achieved very high accuracy and precision on image classifications [5]. 

Based on the excellent performance of DL and convolutional neural network methods in various image classification tasks, this paper aims at evaluating CNN-based MRI feature extraction for the automatic classification of Alzheimer’s diseases. CNN-based models are developed as a DL method to diagnose Alzheimer’s diseases on MRI images with three different classifiers (Softmax, SVM, and RF) and the model’s performance was compared between fully connected layers. The research objectives are to answer the following research questions. (1) Is the pre-train DL CNN approach ResNet50 used in this study useful for the classification of Alzheimer’s diseases in MRI brain images? (2) Which classifier used with pre-trained CNN will give us better classification performance: Softmax, SVM, or RF?

The rest of this paper is organized as follows: Section 2 reviews the previous studies of AD diagnosis and classification, Section 3 presents the methodology, describing how to build and evaluate the proposed CNN model, Section 4 provides the experimental and evaluation results, and, finally, Section 5 concludes the paper and discusses future work.

## 2. Related Work

Several studies have proposed ِAD diagnosis and detection systems that utilize a variety of classification techniques. This section contains a review of recent studies that used conventional ML and DL approaches in AD diagnosis and detection systems. 

Some of the previous studies on Alzheimer’s disease diagnosis have applied conventional machine-learning techniques. They are focused on developing models to analyze the anatomical or structural brain images such as MRI and brain functionality to detect any defect or disorders. In addition, it considered segmentation tasks as classification issues and heavily depended on manually designed features and feature representations as to the voxel, region, or patch-based methods. It required several expert segmented images to train classification models, and that takes a longer time.

Liu et al. (2016) [6] proposed an inherent structure-based multiview learning (ISML) method for AD/MCI classification. The proposed method consists of three stages: (1) multiview feature extraction using multiple templates and using gray matter (GM) tissues as tissue-segmented brain image for feature extraction, (2) subclass clustering-based feature selection through using voxel selection that improving the power of features, and (3) using SVM-based ensemble classification. They evaluated the efficiency of the proposed method on the MRI baseline dataset consisting of 549 subjects (70 AD and 30 Normal Control—NC) provided by the ADNI (http://adni.loni.usc.edu/, accessed on 5 February 2022) database [7]. The experiment result shows that the proposed ISML method obtains an accuracy of 93.83% and specificity of 95.69%, and sensitivity of 92.78% for AD vs. NC.

In another study, Krashenyi et al. (2016) [8] proposed an AD classification approach based on fuzzy logic. Their classification technique is based on multimodal data PET and MRI data. The dataset consists of 70 AD, 111 MCI, and 68 NC subjects provided by the ADNI database. The proposed approach consists of three stages: (1) image pre-processing, including MRI/PET normalization and MRI data segmented into white matter (WM) and grey matter (GM), then they used a voxel selection procedure to reduce low-activated voxels; (2) feature selection is based on ROI, and then they apply t-test as statistical tests for feature ranking and selection to reduce the number of ROI; and (3) do a fuzzy classification using the c-means algorithm. The classification performance of the proposed approach has been used under the receiver operating characteristic (AUC), while the regions with the highest AUC area should be defined in the PET and MRI images as the optimal number of regions. The highest classification performance achieved with a combination of features (7 MRI and 35 PET) is AUC = 94.01%. The experiment result shows that the proposed approach obtains 89.59% accuracy, 92.2% specificity, and 93.27% sensitivity for AD vs. NC.

Lazli et al. (2018) [9] proposed an AD computer-aided diagnosis (CAD) system to distinguish between AD cases and normal control cases and evaluate the tissue volume of MRI and PET images. The proposed approach consists of two processes: segmentation and classification. First, they used fuzzy possibilistic tissue segmentation as a hybrid of the fuzzy c-means (FCM) and a possibilistic c-means (PCM) segmentation processes. Then in the classification process, they used SVM classifiers with different types of kernels (linear, polynomial, and RBF) to decide the final diagnosis (AD or NC). The proposed approach was tested on MRI and PET images that consisted of 45 AD subjects and 50 healthy subjects provided by the ADNI database. The classification performance of the proposed approach has been evaluated with the leave-one-out cross-validation method. The experiment showed that the proposed solution obtains better accuracy, sensitivity, and specificity compared to the other three approaches, FCM, PCM, and VAF [10] (Voxels-As-Features), and achieved a higher accuracy rate of 75% for MRI and 73% for PET images.

The similar work by Thulasi N P and Varghese (2018) [11] proposed a diagnosis system of Alzheimer’s disease based on image processing techniques and SVM classifiers. The proposed approach was trained and tested on a small MRI scanning dataset consisting of 100 subjects (70 AD and 30 NC) provided by the ADNI database. The proposed solution consisted of two phases: feature extraction/selection and classification. In the first phase, the authors used speeded-up robust features (SURF) to extract the key points of the corresponding MRI images, then the gray level co-occurrence matrix (GLCM) was used for feature extraction. In the classification phase, they used the support vector machines (SVM) to classify MRI images to AD or normal controls.

Recently, many improvements have been observed in the research field of AD diagnoses/classification using DL techniques. In opposition to conventional ML methods, DL methods are able to extract/select special features automatically from a raw dataset with higher performance results achieved. Liu et al. (2015) [12] studied Alzheimer’s disease classification using multi-modality data MRI and PET scans from the ADNI dataset. They proposed a novel diagnostic framework to aid AD diagnosis by using DL architecture. To extract complementary information from multimodal neuroimaging data (MRI and PET), their framework uses a stacked auto-encoder SAE and a zero-mask strategy for data fusion. It also uses a Softmax logistic regressor as a classifier. The results show that based on MRI and PET ADNI, this framework outperformed with 91.4% accuracy. However, when PET data is not available and MRI is the only input, this percentage reduces to 82.6%. 

Korolev et al. (2017) [13] apply two different 3D CNN approaches (3D-VGGNet and 3D-ResNet) with Softmax nonlinearity for classification. They use the ADNI dataset of 3D structural MRI brain scans. The result shows that the accuracy of AD/CN classification reaches 79% for Voxnet and 80% for ResNet. In addition, their algorithms are simpler to implement and do not need the manual extraction step.

In recent evidence, Gunawardena et al. (2017) [14] proposed a simple, convolutional neural network for AD pre-detection. Their study consists of two experiments that use MRI scans provided by ADNI. First, they use the SVM classifier as the most common detection method. This decision is based on their assumption that a successful AD detection method can be successfully applied to AD pre-detection. The SVM classifier obtains 84.41% accuracy, 95.3% sensitivity, and 71.4% specificity in the first experiment. For the second experiment, they utilized the proposed CNN model. They tested the CNN model with different datasets and different image segmentation methods through the six evaluation processes. The best image segmentation method was extended ROI without detecting edges, which obtained the best and highest accuracy rate (96%), with 96% sensitivity and 98% specificity. 

Lan Lin et al. (2018) [15] proposed a new classification method that automatically differentiates patients with AD from HC based on MRI data. The feature was extracted from the pre-trained convolutional neural network (CNN) using AlexNet, as well as feature selection based on principal component analysis (PCA) and sequential feature selection (SFS). While they adopt a support vector machine (SVM) to evaluate the classification accuracy, the results show that a high classification accuracy for AD/CN classification reaches 90%. 

Another related work is found in the study elaborated by Bäckström et al. (2018) [16]. Their work was focused on proposing a novel and effective three-dimensional convolutional network (3D ConvNet) architecture to achieve high performance for the detection of AD. The proposed 3D ConvNet consisted of five convolutional layers for feature extraction, followed by three fully connected layers for AD/NC classification. In addition, the study focused on the impact of the following factors on the performance of AD classification: hyper-parameter selection, pre-processing, data partitioning, and dataset size. They obtained a dataset from ADNI consisting of 430 subjects (199 AD and 141 NC). MRI scans were randomly partitioned into three subsets, with 60% in the training set, 20% in the validation set, and 20% in the test set. The results showed that the proposed method achieved a 98.74% accuracy rate for detecting AD vs. CN.

On the other hand, Huanhuan et al. (2019) [5] proposed an ensemble learning method for the early diagnosis of AD by using convolutional neural networks (ConvNets) as a DL technique based on MRI scans. They obtained a dataset consisting of 615 MRI images that were split into 179 AD, 254 MCI, and 182 NC in NifTI format from ADNI. They resized the MRI images to 224 × 224 and grouped them into WM and GM. Only 20 slices were selected as the data from GM and WM and sent to the DL model ConvNet for training. To enhance the classification process, the researchers used ensemble learning methods after the convolutional operations. They selected ResNet50, NASNet, and MobileNet as the combined base classifiers for the early diagnosis of AD. The results show that the proposed method obtained accuracy rates of 98.59 % for AD vs. NC, 97.65% for AD vs. MCI, and 88.37% for MCI vs. NC.

The study by Rallabandi et al. (2020) [17] proposed a model for early diagnosis and classification of AD and MCI from elderly cognitive normal, as well as the prediction and diagnosis of early and late MCI individuals. The dataset consists of 1167 whole-brain magnetic resonance imaging subjects, 371 NC, 328 early MCI, 169 late MCI, and 284 AD, provided by the ADNI database. They used FreeSurfer analysis for each individual scan to extract 68 features of the cortical thickness and utilized these features for building the model. They further tested scans using various machine learning methods (non-linear SVM (RBF kernel), naive Bayesian, K-nearest neighborhood, random forest, decision tree, and linear SVM). The non-linear SVM classifier with radial basis function showed the highest specificity, sensitivity, F-score, Matthew’s correlation coefficient, and kappa-statistic, receiver operating characteristic area under the curve (ROC AUC), as well as 75% accuracy in classifying all four groups using 10-fold cross-validation.

Table 1 below summarizes the reviewed studies and shows a comparison between them based on (1) dataset (dataset name, image modality, and size of the dataset that was used), (2) methodology (feature selection and classifier), and (3) performance evaluation results.

Considering the above, to the best of our knowledge no study has focused on evaluating CNN-based MRI feature extraction using different classifiers. Thus, the aim of this paper is to analyze CNN-based MRI feature extraction for automatic classification of patients with Alzheimer’s disease using pretrained CNN ResNet-50 with SVM, RF, and Softmax.

## 3. Materials and Methods

**Figure 1:**
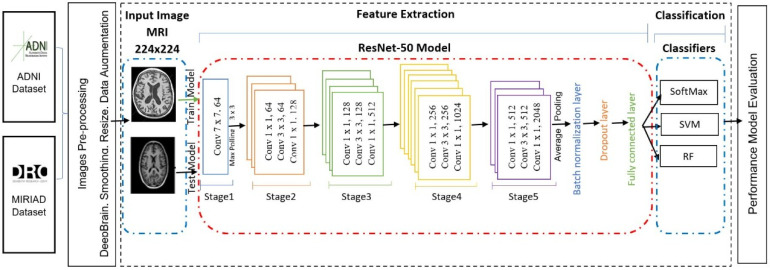
The general structure of the proposed approach.

The main aim of this paper is to investigate and enhance the classification performance of MRI images for the early diagnosis of AD through DL and CNN. Thus, this paper proposes to build and evaluate a disease diagnosis approach based on a CNN DL technique based on MRI feature extraction for the automatic classification of AD using three different classifiers, SVM, RF, and Softmax. Figure 1 shows the general structure of the proposed approach.

**Figure 2:**
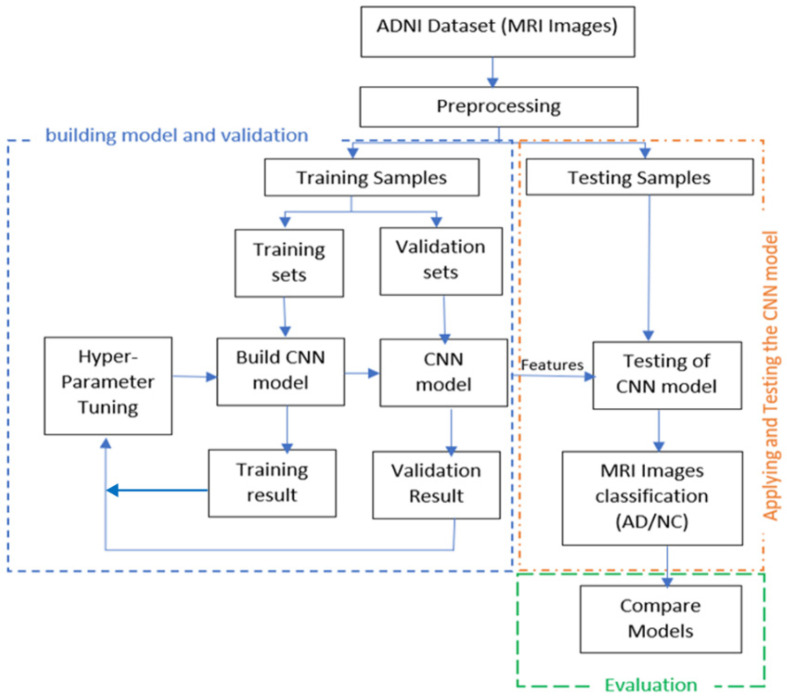
The detailed steps of the proposed solution.

In this work, we will start by building and validating a CNN model for feature extraction and classification. The validated model will then be used in experiments to evaluate the model through analyzing the features extracted by CNN (ResNet) from the fully connected layer. Three of the most well-known conventional ML classifiers will be applied (SVM, RF, and Softmax) for each set of features and for evaluating the results, where SVM and RF are the most common classification techniques used for AD classification based on our literature review. To build an AD diagnosis approach, the methodology goes through the following stages: first, the MRI data collection stage. In the second stage, the image pre-processing, we resized each MRI image to a suitable size for the CNN model. After that, we employed the pre-trained convolution neural networks ResNet50 to extract MRI image features and utilize them in the following classification stage in the feature’s extraction stage. We use three different classifiers, SoftMax, SVM, and RF, in the classification stage. Finally, we looked at the different results, analyzed the efficiency and the effectiveness of each approach using the evaluation metrics, and compared our results with recent studies results. Figure 2 illustrates the detailed steps of the solution. 

### 3.1. MRI Dataset

This study will use two public datasets, the Alzheimer’s Disease Neuroimaging Initiative (ADNI) [7] and Minimal Interval Resonance Imaging in Alzheimer’s Disease (MIRIAD) [18]. The ADNI [7] was launched in 2003 by the National Institute of Biomedical Imaging and Bioengineering as a non-profit organization led by principal investigator Michael W. Weiner, MD. The initial goal of ADNI is to evaluate the progression of early Alzheimer’s disease. The ADNI-1 Dataset consists of 1.5 T T1-weighted MRI images with 128 sagittal slices, typically 256 × 256 matrices with a voxel size of approximately 1.33 mm × 1 mm × 1 mm). The dataset is encompassing 741 subjects divided into Alzheimer’s disease (AD) and normal control (NC). This dataset consists of 314 AD scans and 427 NC [7]. The MIRIAD dataset is a publicity available scan database of MRI brain scans consisting of 46 Alzheimer’s patients and 23 normal control cases. Many scans were collected from each participant at intervals between 2 weeks and 2 years, and the study was designed to examine the feasibility of using MRI scans as an outcome measure for clinical trials of Alzheimer’s therapies. It includes a total of 708 scans. Three-dimensional T1-weighted images were acquired with an IR-FSPGR (inversion recovery prepared fast spoiled gradient recalled) sequence, field of view 24 cm, 256 × 256 matrix, 124 1.5 mm coronal partitions, TR 15 ms, TE 5.4 ms, flip angle 15°, and TI 650 ms [18]. In both datasets, images from AD patients did not specify AD degrees. In our experiments, multiple images from one patient are treated independently, as if for different patients.

**Figure 3:**
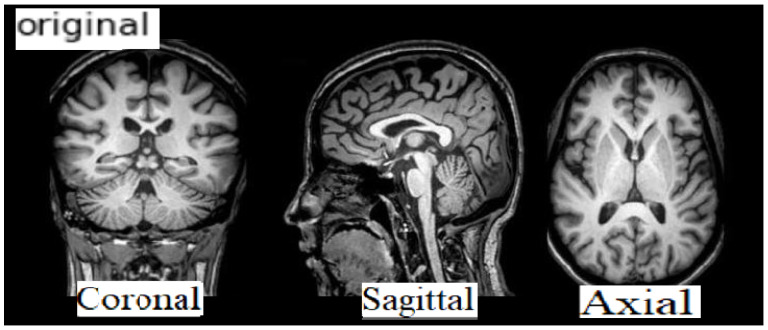
MRI Imaging Planes.

**Figure 4:**
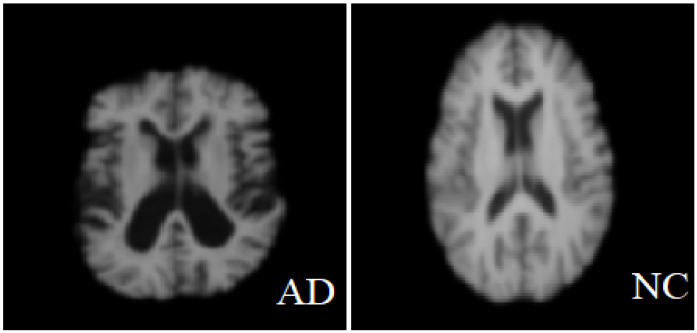
Normal brain vs. brain affected with Alzheimer’s.

The data format is NIFTI and the file extension is (.nii). MRI data provide details of the brain and visualize the anatomy in all three planes: axial, sagittal, and coronal (see Figure 3 below). Figure 4 shows a comparison between a healthy brain (NC brain) and an AD brain of axial planes [18].

### 3.2. Data Pre-Processing

The pre-processing phase of the MRI datasets aims to transform the data into a more optimal representation to match the pre-trained CNN’s input size requirements. First, we extracted the brain from MRI 3D images by removing the skull from the image and eliminating noise for improving the model performance. Then, applying the smoothing technique of MRI is often used to reduce noise within an image and produce a less pixelated image. We have smoothed our MRI images with a 4 mm FWHM Gaussian filter, while FWHM is the width of the kernel. Moreover, the ResNet architecture uses input images 224 × 224 pixels in size, meaning that each input MRI image in our CNN model resized to 224 × 224 pixels before being fed into the model. 

### 3.3. CNN Model

The architecture of the proposed pre-trained CNN model (ResNet-50 [19]) consists of five Conv blocks stages, pooling layers, and the fully connected (FC) layer. Convolutional and pooling layers are used for feature extraction, while the fully connected layers are used for the image classification stage. Feature extraction in CNN uses local connections for local features detected and pooling for merging similar local features to be one feature. Meanwhile, FC layers are used to compute the output for each input MRI image. In addition, for optimizing the classification task, the FC layers can be replaced by other classifiers, such as SVM or RF.

After the data collection and image pre-processing stage, the dataset is divided into three sets: training, validation, and a testing set. Since we have small datasets, we used the data augmentation technique, which helps to increase the number of samples in our training dataset and this has expanded the number of images to 741 for ADNI [7] and 708 for MIRIAD [18]. The training set (a labeled dataset) trains the CNN model on a particular task, such as feature extraction, where the CNN model will generate MRI features vectors from the fully connected layer. After that, the features vectors are entered into three different classifiers. The validation set provides an impartial evaluation of a model fit on the training dataset while tuning the model. The test set was used to evaluate the ResNet50-Softmax, ResNet50-SVM, and ResNet50-RF model approaches.

We apply a pre-trained CNN called ResNet-50 using Tensorflow [20] and Keras [21] applications to the MRI images instead of training a CNN from scratch, which requires a huge dataset. In addition, this helps to avoid the overfitting problem caused by the small dataset. We selected the ResNet-50 model because it is arguably the most groundbreaking work in the computer vision/DL community in the last 5 years. ResNet makes it possible to train hundreds of layers that go deeper and deeper and still achieve good performance. It won the 2015 ImageNet Large Scale Visual Recognition Challenge (ILSVRC) and it outperformed all prior competitors and won the challenge by reducing the top-five error to 3.57%. Our study used ResNet-50 with three classifiers, Softmax, SVM, and RF, to determine which one performs better with the ResNet-50 model. 

In the following paragraphs, there will be a brief description of the methodology CNN model of each set of layers.

#### 3.3.1. Convolutional Layer

The convolutional layer is the essential part and the core building block of the DL CNN. It is responsible for the feature extraction process, while its output sets of 2D matrices are called feature maps. Each convolutional layer consists of a fixed number of filters that act as feature detectors and extract the features by convolving the input image with these filters. The size of the filters is chosen in ResNet50 (7 × 7), (1 × 1), and (3 × 3). During the training process, each filter acquires the ability to detect the analyzed images’ low-level features, such as colors, edges, blobs, and corners.

#### 3.3.2. Pooling Layer

The pooling layers [22] are places after the convolutional layers (Conv). The subsampling layer is responsible for decreasing the size of the feature maps that produce the convolutional layers. Max pooling is the most popular pooling operation that reduces the feature maps by reducing the small region in the image with the maximum value in the region. The max-pooling process is based on the partition of the images into sets of 2 × 2 non-overlapping regions. The maximum value from every region is taken. The 2 × 2 pooling layer reduces the size of the feature map by four times. 

The process of max pooling is performed to avoid overfitting by providing an abstract of the image representation regions. In addition, it minimizes the computational cost by decreasing the number of parameters. Furthermore, the average pooling layer is another type of pooling. This layer acts as max pooling, but it calculates 2 × 2 rectangles’ averages to create a subsampled image instead of taking the maximum value.

#### 3.3.3. Batch Normalization Layer

The batch normalization layer [23] is used to normalize the convolution layer’s output by setting the batch’s mean to 0 and the variance to 1. This technique speeds up the training process, using higher learning rates. Moreover, it prevents the gradients of the model from vanishing during backpropagation. In addition, DL models with batch normalization layers are more robust against improper weights initialization. 

#### 3.3.4. Dropout Layer

The dropout layer [24] is used to avoid overfitting phenomena. This technique is based on a mechanism where, during the training, neurons are randomly removed. The dropout rate parameter controls the number of removed neurons, which decides the likelihood of neuron removal. The neurons are removed only during the training process.

#### 3.3.5. Fully Connected Layer

The fully connecting layer is the last layer in the ResNet50 network. It acts as a classifier, and its function is to connect the layers in the network and give the final result of the classification. Usually, it is followed by the final layer with a normalized exponential function (Softmax). This layer has been modified to fine-tune the ResNet50 for the classification of Alzheimer’s disease.

### 3.4. MRI Image Classification 

The FC layers of CNN can be replaced by other classifiers, for example, based on logistic regression or SVMs, which are optimized for the task of classification. In this project, we will evaluate CNN with Softmax, SVM, and RF classifier.

#### 3.4.1. Softmax Classification Layer

In general, in the last layer of CNN architecture is the Softmax function used to classify the labeled data and calculate the probability of each ground-truth label of outputs between 0 and 1, and output values converted to perceptible values. The formula of the Softmax function is given by the following equation [25]:

(1) $$f\left(x\right)i=\frac{e^{zj}}{\sum_{n=1}^{N}e^{zk}}for\;j=1,\;\ldots \ldots ,N,$$

where *N* is denoted as the dimension of random values (*x*), which are converted to the meaningful values between 0 and 1 by the Softmax function *f*(*x*).

#### 3.4.2. SVM Classification

We will replace the final FC layers by SVM classifier with a number of splits (folds number = 10 and seed = 7). SVMs are often used for binary image classification, AD vs. NC, and they have achieved noteworthy results in real-life problems. In addition, using the RBF kernel, the SVM classifier generates a nonlinear classifier that can map the original dataset to the higher dimensional space by generating linear data. This is shown in the equation below, where input vectors are shown by x and y, the squared Euclidean distance between *x* and *y* vectors is shown by ${\left|\left|x-y\right|\right|}^{2}$, and the kernel parameter is shown by $\sigma^{2}$ [25]:

(2) $$k\left(x,y\right)=\exp\left(-\frac{{\left|\left|x-y\right|\right|}^{2}}{2\sigma^{2}}\right),$$

#### 3.4.3. Random Forest

Random forest (RF) is a technique for reducing the variance of an estimated prediction function. RF is a substantial modification of bagging that builds a large collection of de-correlated trees and then averages them. The essential idea in bagging is to average many noisy but approximately unbiased models and reduce the variance. Trees are ideal candidates for bagging since they can capture complex interactions [26]. It can be used for classification and regression. When used for classification, a random forest obtains a class vote from each tree and then classifies using a majority vote. When used for regression, the predictions from each tree at target point x are simply averaged. In our study, we used RF for classification with number estimator = 20 while the default = 100 and it can be change from 1–100 after trying several values 20 gives us the best result. 

On many problems, random forests’ performances are much like boosting, and they are simpler to train and tune. Consequently, random forests are popular, and are implemented in a variety of packages [26]. 

### 3.5. Performance Evaluation Metrics 

The most important performance indicator (accuracy, ACC) of the AD diagnosis is used to measure the ResNet50-Softmax, ResNet-SVM, and ResNet-RF performance models. In addition, sensitivity (*SEN*) and specificity (*SPE*) are performance indicators. The true positives (*TP*) refer to the classifier’s positive tuples that were correctly labeled. Let TP be the number of true positives. The false positives (*FP*) are the negative tuples that were incorrectly labeled as positive. Let FP be the number of false positives. The true negative (*TN*) are the negative tuples that the classifier correctly labeled. Let TN be the number of true negatives. The false negatives (*FN*) are the positive tuples that were mislabeled as negative. Let FN be the number of false negatives.

- Accuracy (*ACC*): the percentage of the number of records classified correctly versus the total records shown in the equation below:

(3) ACC=(TP+TN)/(TP+TN+FP+FN),

- Sensitivity (*SEN*)/Recall shows the percentage of the number of records identified correctly over the total number of AD subjects, as shown in the equation below:

(4) SEN=TP/(TP+FN),

- Specificity (*SPE*): the percentage of the number of records. Normal control is divided by the total number of normal nodes, as shown in the equation below:

(5) SPE=TP/(TP+FP),

- *F_measure_*: a measure of a test’s accuracy:

(6) $$F_{measure}=2*\frac{\left(precision\;*\;recall\right)}{\left(precision+recall\right)}$$

## 4. Experiments and Results

This section describes the conducted experiment and its setup, followed by our experiment’s results. We will first give a brief description of the experiment’s setup, which includes software and hardware settings, followed by the results of model training and validation. The third subsection is related to the obtained results when applying the CNN model for feature extraction with the three classifiers (Softmax, SVM, and RF). Finally, we will compare the obtained results in the proposed approach with those of other methods. 

### 4.1. Experimental Setup

The experiments were conducted using the Google Colaboratory Pro [27] platform (Colab Pro) as a Python development environment. It is a cloud service provided by Google that allows users to write and execute Python codes in a hosted GPU. We used DL Python libraries TensorFlow [20], Keras [21], Scikit-learn [21], Numpy [21], and OpenCV [28] for developing the proposed solution. In addition, we used Nibabel [29], Nilearn [30], and DeepBrain [31] as Python libraries for neuroimaging data (MRI) analysis. This study employed an ADNI [7] dataset with the NIFTI format of MRI scans and focused on coronal plane visualization of brain anatomy. A coronal plane is an x-z plane perpendicular to the ground, which (in humans) separates the anterior from the posterior. Studies show that using the coronal plane is more effective [32].

The dataset consists of 741 subjects [AD:427 and, NC: 314]. As a pre-processing stage, for ResNet-50 we needed to resize all MRI images to 224 × 224 and convert them to RGB. 

### 4.2. The Results of Model Training and Validation

In our study, the dataset was randomly partitioning into 75:15:10, 75% for the training, 15% for validation, and 10% for testing. Table 2 below shows the details of the dataset. 

The proposed CNN model structure is the same as that of the ResNet50 model, with some modifications that were made to avoid overfitting and enhancing the model performance. After the last convolution layer and after each fully connected layer, a batch normalization layer was added to normalize the output. One dropout layer was added before the classifier and after the last fully connected layer to avoid overfitting phenomena, while the dropout rate was set to 0.5. The ResNet-50 network was trained using the stochastic gradient descent (SGD) optimizer with the learning rate to 0.0004 and momentum to 0.9. The batch size was set to 10 for training and validation sets, while batch size equals sample number in the testing set. We set epoch to 100, while it is a hyperparameter predefined before training a model.

**Figure 5:**
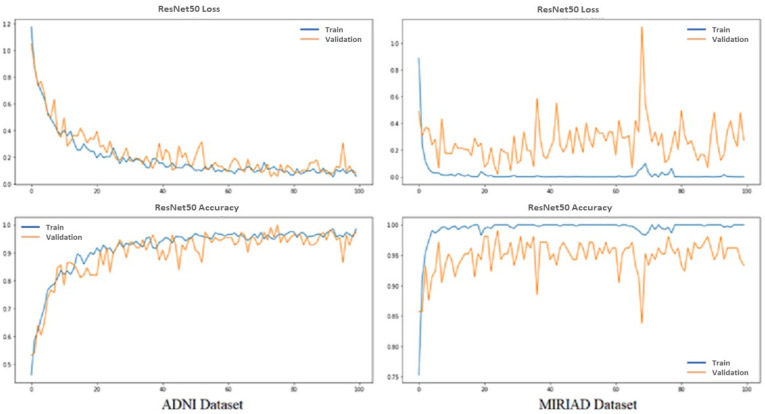
Training and validation performance of ResNet50-Softmax.

We evaluated the model based on the accuracy and categorical cross-entropy (loss) of classification AD and normal MRI images. Loss functions are intended to compute the quantity that a model should seek to minimize during training. Figure 5 shows the performance of the proposed pretrained CNN model ResNet-50 from training and validation for the ADNI and MIRIAD datasets. Top graphs show loss vs. epochs; meanwhile, down graphs show accuracy vs. epochs; red from training and orange from validation results, while epochs equal 100.

### 4.3. Classification Result Evaluation or Performance Analysis

To answer our first and second research questions, different experiments were conducted using three different classifiers (Softmax, SVM, and RF). Thus, in our experiments, we evaluated the proposed model’s classification performance using Softmax, SVM, and RF classifiers with the ADNI [7] dataset and MIRIAD [18] dataset.

The experiment aims at determining the most accurate approach for the AD diagnostic pre-train model ResNet50. First, we apply transfer learning on ResNet50 using Softmax in the classifier layer. After that, the proposed approaches (ResNet50-Softmax, ResNet50-SVM, and ResNet50-RF) were tested on the ADNI and MIRIAD datasets. Results showed that the model with the Softmax classifier outperforms SVM and RF in all performance measures. Table 3 below shows the accuracy, specificity, sensitivity, and F-measure of each classifier on both datasets.

**Figure 6:**
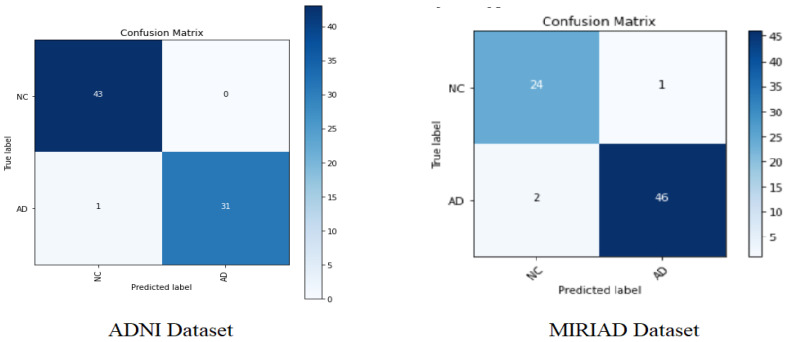
Confusion matrix obtained on the test dataset with Resnet50-Softmax.

Since Softmax has the best results over the other classifiers (RF and SVM) for both datasets, we will investigate more in evaluating this classifier’s performance in terms of individual classes [AD and NC]. Figure 6 below shows the confusion matrix for the Softmax classifier on the ADNI and MIRIAD datasets. Table 4 and Table 5 show the classification performance results of Softmax in precision, recall, f1-measure, and support, where support represents the number of samples.

From Figure 6 and Table 4 and Table 5, it can be clearly observed that the proposed AD diagnosis model has been shown to be effective, with a favorable AD classification rate (96.875%) and a low false alarm of 3.125% for the ADNI dataset, and with an AD classification rate (95.83%) with a low false alarm of 4.16% for the MIRIAD dataset.

Results also show that the performance is consistent in the three classifiers. This is demonstrated by the accuracy achieved by the Softmax classifiers being the highest. Likewise, SVM comes as the second-best classifier, and RF comes third. This shows that performance of the proposed model is not affected by the dataset.

### 4.4. Comparison with the State-of-the-Art Models

**Figure 7:**
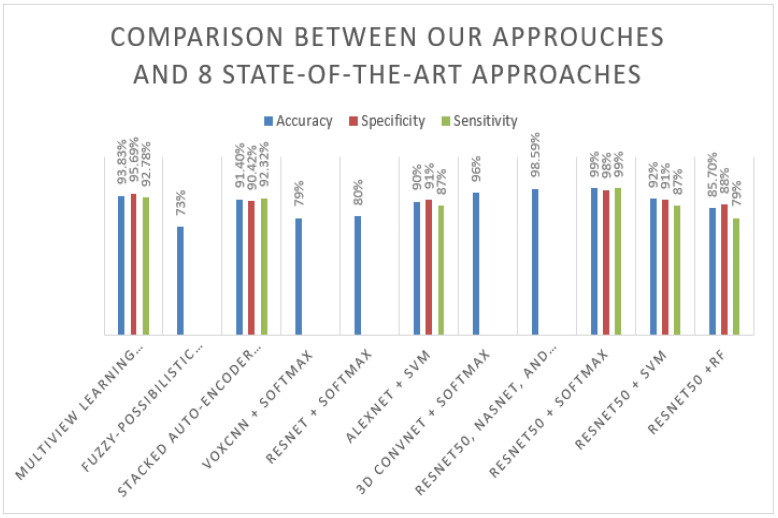
Comparison between our approaches and eight state-of-the-art approaches.

As shown in the previous sections, the proposed model has shown promising results on the ADNI [7] and MIRIAD [18] datasets with three different classifiers, with accuracy, specificity, sensitivity, and F-measure. It gave good results, answering our first research question about the effectiveness of the proposed model needs and evaluating its classification performance compared to other approaches in the literature to assess its effectiveness. To achieve that, we need to compare its performance against some of the state-of-the-art approaches discussed in our literature review. The approaches in the related work section were tested on the MRI of the ADNI [7] dataset. We compared the obtained results on ADNI with our proposed method in all three approaches (ResNet-50 + Softmax, ResNet-50 + SVM, and ResNet-50 + RF). Table 6 shows the results of our proposed model on the ADNI dataset in comparison to the results obtained by other approaches. From the results, it can be clearly seen that the proposed ResNet50-Softmax approach achieved very high performance of 99%, close to the approach result proposed by Huanhuan et al. [5], which combines three pre-trained models, including ResNet50, and achieves 98.59%. In addition, the proposed approach using different techniques such as MRI image smoothing, add batch normalization, and dropout layers that improve the network performance, compared with the ResNet50-Softmax approach proposed by Korolev et al. [13], is one of the proposed approaches using the same pre-train model ResNet50 without add batch normalization, or dropout and achieves only 80%. On the other hand, ResNet50 + SVM obtains a good result compared with [6,9,13,16]; it achieves 92% accuracy. In addition, ResNet + RF outperform three approaches that are the fuzzy-possibilistic tissue segmentation + SVM approach [9], VoxCNN + Softmax, and ResNet50 + Softmax proposed by [13]. Figure 7 below represents the results in the flow chart.

## 5. Conclusions

To conclude, in this paper an Alzheimer’s disease classification model was developed for MRI. The pre-trained convolution neural network (CNN) architecture ResNet-50 was applied for an AD diagnoses system with different approaches (ResNet50 + Softmax, ResNet50 + SVM, and ResNet50 + RF). First, we evaluated the performance for transfer learning from pre-trained CNN ResNet-50 for the classification task using Softmax. After that, we evaluated the performance for ResNet-50 for extracting features and using a support vector machine (SVM) and random forest (RF) for the classification task. This study was conducted on the ADNI MRI and MIRIAD datasets. The results show an accuracy of 99% for ResNet + Sofmax, 92% for ResNet50+SVM, and 85.7% for ResNet50 + RF with the ADNI dataset, while the results of the MIRIAD dataset showed accuracy of 96% for ResNet + Sofmax, 90% for ResNet50 + SVM, and 84.4% for ResNet50 + RF. We compared our model with the state-of-the-art models using the ADNI dataset and the results show that our model ResNet50 + Softmax achieved a higher accuracy than most of the state-of-the-art models. 

## Figures and Tables

**Table 1:** Summary and comparison of the selected recent research.

References	Dataset			Methodology		Evaluation Result
	Name	Modality	Size	Feature Selection	Classifier	
Liu et al. (2016) [6]	ADNI	MRI	549 subjects 70 AD 30 NC	Multiview learning using GM	SVM	AD vs. NC. Accuracy: 93.83% specificity: 95.69% sensitivity: 92.78%
Krashenyi et al. (2016) [8]	ADNI	MRI PET	249 subjects 70 AD 111 MCI 68 NC	ROI + statistical tests (t test)	fuzzy logic using: c-means algorithm	AD vs. NC Accuracy: 89.59% specificity: 92.2% sensitivity: 93.27% AUC= 94.01%.
Lazli et al. (2018) [9]	ADNI	MRI PET	95 subjects 45 AD 50 NC	Fuzzy-Possibilistic Tissue Segmentation	SVM (Linear, Polynomial, and RBF)	AD vs. NC Accuracy: 75% (for MRI), 73% (for PET)
Thulasi N P and Varghese (2018) [11]	ADNI	MRI	100 subjects 70 AD 30 NC	Speeded Up Robust Features (SURF) Gray Level Co-Occurrence Matrix (GLCM)	SVM	-
Liu et al. (2015) [12]	ADNI	MRI PET	758 MRIsubjects 180 AD 160 cMCI 214 ncMCI 204 NC 331 subject Both MR & PET data 85 AD 67 cMCI 102 ncMCI 77 NC	stacked auto-encoder SAE	Softmax logistic regressor	AD vs. NC Accuracy: 91.40% specificity: 90.42% sensitivity: 92.32% MCI vs. NC. Accuracy: 82.10% specificity: 92.32% sensitivity: 60.00%
Korolev et al. (2017) [13]	ADNI	MRI	231 Subjects 50 AD 43 LMCI 77 EMCI 61 NC	3D CNN (VoxCNN & ResNet)	Softmax	AD vs. NC Accuracy: 79% VoxCNN 80% ResNet AUC: 88% VoxCNN 87% ResNet
Gunawardena et al. (2017) [14]	ADNI	MRI	D1: 36 subjects (AD 7, MCI 14, NC 15) > 1615 2D images generated D2: 36 subjects (AD 9, MCI 16, NC 11) > 1743 2D images generated from 3D	CNN	SVM	The best classification accuracy (96%) with (Extended ROI without detecting edges) among the other segmentation methods
Lan Lin et al. (2018) [15]	ADNI	MRI	422 subjects 105 AD 123 MCI 194 NC	CNN (AlexNet)	SVM	AD vs. NC Accuracy: 90% specificity: 91% sensitivity: 87% AD vs. MCI Accuracy: 81% specificity: 88% sensitivity: 70% MCI vs. NC Accuracy: 72% specificity: 74% sensitivity: 69%
Bäckström et al. (2018) [16]	ADNI	MRI	340 subjects 199 AD 141 NC 1198 MRI Scans	3D ConvNet	Softmax	AD vs. NC Accuracy: 98%
Huanhuan et al. (2019) [5]	ADNI	MRI	615 subjects 179 AD 254 MCI 182 NC	ConvNet	ResNet50, NASNet, and MobileNet	AD vs. NC Accuracy: 98.59% AD vs. MCI Accuracy: 97.65% MCI vs. NC Accuracy: 88.37
Rallabandi et al. (2020) [17]	ADNI	MRI	1167 subjects 371 NC 328 EMCI, 169 LMCI 284 AD	FreeSurfer	Non-linear SVM (RBF kernel) Naive Bayesian K-Nearest Neighborhod Random Forest Decision Tree Linear SVM	non-linear SVM classifier showed the highest result in classifying all four groups 77% specificity 75% sensitivity 72% F-score 71% Matthew’s correlation coefficient 69% kappa-statistic 76% (ROC AUC) 75% accuracy

**Table 2:** Datasets details.

Data Set	Size	Training (75%)	Validation (15%)	Testing (10%)
ADNI [7]	741 [AD:427, NC:314]	555	111	75
MIRIAD [18]	708 [AD:466, NC:243]	530	105	73

**Table 3:** Performance of the three classifiers in the proposed model.

Dataset	Classifier Used with ResNet50	Accuracy	Specificity	Sensitivity	F-Measure
ADNI [7]	Softmax	**99%**	**98%**	**99%**	**98%**
	SVM	92%	91%	87%	89%
	RF	85.7%	88%	79%	84%
MIRIAD [18]	Softmax	**96%**	**95%**	**96%**	**97%**
	SVM	90%	91%	87%	87%
	RF	84.8%	84%	73%	79%

**Table 4:** Resnet50-Softmax experiment results with the ADNI dataset.

	Precision	Recall	F1-Score	Support
NC	98%	100%	99%	43
AD	100%	97%	98%	32
Accuracy			99%	75
Macro avg	99%	98%	99%	75
Weighted avg	99%	99%	99%	75

**Table 5:** Resnet50-Softmax experiment results with the MIRIAD dataset.

	Precision	Recall	F1-Score	Support
NC	92%	96%	94%	25
AD	98%	96%	97%	48
Accuracy			96%	73
Macro avg	95%	96%	95%	73
Weighted avg	96%	96%	96%	73

**Table 6:** Comparison of our test performance with eight existing state-of-the-art methods.

Models	Used Approach	Accuracy	Specificity	Sensitivity
Liu et al. (2016) [6]	Multiview learning using GM + SVM	93.83%	95.69%	92.78%
Lazli et al. (2018) [9]	Fuzzy-Possibilistic Tissue Segmentation + SVM	73%	-	-
Liu et al. (2015) [12]	stacked auto-encoder SAE + Softmax	91.40	90.42%	92.32%
Korolev et al. (2017) [13]	VoxCNN + Softmax	79%	-	-
	ResNet + Softmax	80%	-	-
Lan Lin et al. (2018) [15]	AlexNet + SVM	90%	91%	87%
Bäckström et al. (2018) [16]	3D ConvNet + Softmax	96%		
Huanhuan et al. (2019) [5]	ResNet50, NASNet, and MobileNet + Softmax	98.59	-	-
Rallabandi et al. (2020) [17]	Non-linear SVM (RBF kernel)	75%	79%	75%
Proposed model ADNI [7]	ResNet50 + Softmax	99%	98%	99%
	ResNet50 + SVM	92%	91%	87%
	ResNet50 + RF	85.7%	88%	79%

## Data Availability

Not applicable.
